# A Simple Approach to Sample Preparation for Accurate and Reproducible Gas Phase Breakthrough Analysis of Adsorbent Materials

**DOI:** 10.3390/mps9030080

**Published:** 2026-05-29

**Authors:** Daniel A. Corbin, Christopher J. Breshike, Michael R. Papantonakis, R. Andrew McGill

**Affiliations:** US Naval Research Laboratory, 4555 Overlook Ave SW, Washington, DC 20375, USA

**Keywords:** breakthrough, breakthrough analysis, BTA, sorbent, adsorbent, metal–organic framework, MOF

## Abstract

A simple approach to sample preparation for gas phase breakthrough analysis of adsorbent materials such as metal–organic frameworks is reported. To circumvent issues related to particle size, MOF powders are coated onto glass beads using only the adhesion forces between the glass surface and the particles themselves. These coatings are sufficiently stable for the coated beads to be packed into columns and used for breakthrough measurements of the pure solids. Samples prepared in this manner are compared to analogous samples coated using a binder to attach the MOF to the surface of the beads. In many cases, the approach reported here achieves higher uptake capacities and longer breakthrough times than when a binder is used, presumably because the binder partially clogs the porous structure of the MOF. In addition, an example is discussed that highlights the possibility of a reaction between the sorbent and the binder, highlighting the advantage of a simplified sample preparation method that does not require additional chemical additives.

## 1. Introduction

Sorbent materials are becoming increasingly important for gas separation and purification, including natural gas purification [1,2,3], CO_2_ capture from air and industrial effluents [1,4,5,6], and hazardous chemical capture for air filtration [7,8,9,10]. To address these needs, significant resources have been dedicated to the development of metal–organic frameworks (MOFs) as sorbents. In part, these materials are desirable for their high surface area and chemical diversity [11,12,13], which allows the sorbent to be tailored to a specific application. Their highly ordered periodic structures [13] also facilitate fundamental investigations of their structure–property relationships to understand and improve their performance through iterative design.

To evaluate the separation capabilities of these materials, dynamic breakthrough analysis has been recommended [7,10,14,15]. In this technique, a bed or column of the sorbent material is prepared and exposed to a continuous stream of a challenge gas, which may be composed of one or several chemicals in makeup gas such as dry air, humid air, or nitrogen. As the gas stream flows through the sorbent, it selectively captures one or more components until it becomes saturated and the challenge gas “breaks through” the sorbent column. This process is monitored by measurement of the effluent gas mixture, which allows key performance metrics for the sorbent to be determined such as total uptake capacity, breakthrough time, ability to separate gases, and adsorption kinetics.

**Figure 1:**
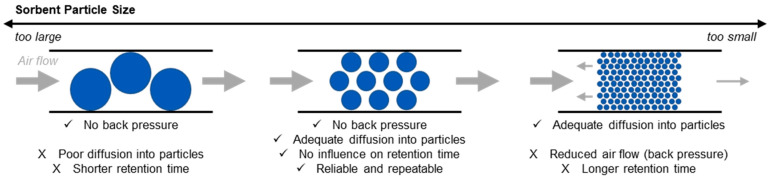
Diagram showing the effect of sorbent particle size on breakthrough measurements. Grey arrows indicate the direction and magnitude of airflow.

Consideration of sorbent particle size is crucial for obtaining reliable data. If the sorbent particles are too large or too small, the challenge gas may not interact with the sorbent optimally, leading to artificial effects that obscure the sorbent’s true properties (Figure 1). For example, if the sorbent particles are excessively large, the gas stream may not diffuse well into the sorbent. As a result, the gas will experience shorter residence times and faster breakthrough. Conversely, excessively small particles (e.g., fine powders) can also have deleterious effects when they pack too tightly and restrict airflow (i.e., cause back pressure). The ultimate effect in this case is delayed breakthrough [16,17,18]. Therefore, sample preparation and sorbent particle size must be considered to ensure that the measured data are truly representative of the sorbent’s fundamental properties.

To this end, several approaches have been reported for shaping sorbent powders into larger particles such as pellets, granules, and beads [18,19,20,21]. These techniques commonly involve compressing the sorbent powders into larger form-factors with high pressure, or alternatively forming agglomerates or extrusions that are held together with a binder. However, specialized equipment may be required, and care must still be taken to ensure that particle size and size distribution remain consistent across different samples. In situ growth of MOF sorbents on the surface of a support material has also been reported for microextraction and separation applications [22,23,24,25,26], but this approach may not be appropriate for some sorbent materials (ex. activated carbon). Moreover, in situ growth often requires custom modification of the support surface to enable attachment of the MOF, which may vary from one material to another and further complicates the shaping process [23,26]. To simplify the shaping process, Alhashem et al. recently reported an interesting approach for coating MOF powders onto glass beads using a binder [18]. This approach was demonstrated with several different MOF sorbents and was shown to improve breakthrough experiments by reducing the buildup of back pressure in the sorbent columns. The use of glass beads as a substrate also meant that minimal preparation of the sorbent was required prior to coating, and uniform particle sizes could easily be achieved across different samples.

Unfortunately, many of these shaping processes alter material properties by reducing the surface area of the sorbent. For example, when powders are compressed into pellets, the applied pressure often causes partial collapse of the sorbent crystal structure, leading to reduced surface areas. The use of a binder can also reduce the surface area of the sorbent, since it can penetrate into the porous structure and partially block pores [19]. Even the work of Alhashem et al. showed a 10% to 27% reduction in surface area depending on the MOF under study [18]. Since these changes in surface area can be expected to impact the performance of the sorbent, we wondered whether another approach might be possible to enable more accurate measurements of the sorbent properties.

In particular, we were motivated by other work investigating particle deposition on various surfaces such as steel, glass, and polyethylene [27,28,29]. It was found that particles in the range of 1–30 μm could be sieved onto glass substrates and held in place by simple adhesive forces, such that the samples could be handled under normal conditions without loss of the particles [27]. Several adhesive forces exist that can contribute to this behavior, including electrostatic (due to charge building up on an insulating surface) and van der Waals forces, which become increasingly important as particle size decreases. For particles in the range of ~10 μm and smaller, these adhesive forces can exceed the gravitational force by several orders of magnitude [30], though the exact nature of the surface interaction and the relationship of particle size to adhesive strength depends on both the surface and particle material. In addition, surface roughness can play a role by impacting the contact area between a particle and the surface, although the precise effect depends both on the size of the surface features as well as the size of the particles [31]. Nevertheless, we wondered whether adhesive forces might be sufficient to coat MOF powders directly onto glass beads such that the binder could be eliminated in the coating process. This approach would enable measurement of the pristine sorbent by breakthrough analysis while maintaining sufficient void volume within the sample column to allow unimpeded airflow.

## 2. Materials & Methods

Additional data related to this manuscript are available in the electronic Supplementary Materials. With the exception of MOF-808-Ac, all MOF sorbents were purchased and used as received from Strem (Newburyport, MA, USA). MOF-808-Ac was prepared according to a literature procedure [32]. Glass beads (1 mm diameter, Milwaukee, WI, USA) for sample coating were purchased from Sigma Aldrich (Z273619).

Coated beads were prepared as follows: for a standard column, approximately 40 mg of the MOF was combined with 1 g of glass beads (1 mm diameter) in a glass scintillation vial. The size of the vial was minimized to reduced sample loss on the sides of the vial. The mixture was vortexed extensively to ensure complete coverage of the glass beads with the MOF powder.

Sample columns were prepared by first plugging one end of a glass tube (4 mm ID, 6 mm OD, McMaster-Carr, Elmhurst, IL, USA) with a wad of glass wool (10 mg). Next, 250 μm glass beads (Cospheric SLGMS-2.5, 200 mg, Somis, CA, USA) were added on top of the glass wool to create a flat surface for the sample. The coated beads were added to the column and settled by gently tapping the column, with care being taken to exclude any loose powder. The mass of beads used was subtracted from the weight of the column before and after addition to calculate the mass of the MOF sample added to the column. Once the sample was fully settled, another layer of 250 μm glass beads (200 mg) was added to the top of the column, and the open end was plugged with another wad of glass wool. For a column prepared as described here, the typical mass of the MOF was approximately 25 mg.

Breakthrough measurements were performed on a Breakthrough Analyzer produced by Surface Measurement Systems (Allentown, PA, USA). Water vapor was generated by bubbling dry nitrogen through a water bath to generate a saturated vapor stream, which was then diluted to the desired concentration (relative humidity level) by mixing with a second stream of dry nitrogen. Column breakthrough was monitored using a humidity sensor (Rotronic HygroClip2, Bassersdorf, Switzerland) at the column outlet. All measurements were performed at a flow rate of 5 sccm and a relative humidity of 20% at 25 °C. Prior to measurement, columns were conditioned by heating to 150 °C for 2 h under 50 sccm dry nitrogen flow, followed by 2 h with 50 sccm nitrogen at 25 °C to ensure temperature equilibration prior to the measurement.

A detailed explanation of the data analysis performed in this work is provided in the Supplementary Materials along with an example. Briefly, all data analysis was performed in Origin 2024. Data analysis was performed by normalizing all data to the saturated signal at the end of a breakthrough measurement, which corresponded to the concentration of the challenge vapor stream. The value for the saturated signal was determined by averaging the data for the last 20 min of the experiment. In addition, the baseline of each measurement was corrected to ensure accurate curve integration by setting the baseline value to the average value of the signal for the 20 min preceding introduction of the challenge vapor.

Uptake capacity was determined by integrating the area under the breakthrough curve for a given sorbent and a blank column prepared with a similar mass of uncoated glass beads. The difference between these two integrations was determined as a percentage and represents the percentage of vapor that was adsorbed during the experiment. Combined with the concentration of the challenge vapor, this value was used to determine the total quantity of vapor that was adsorbed by the sorbent column. This value was divided by the mass of sorbent used in the experiment to determine the uptake capacity of the sorbent.

Breakthrough time was determined by measuring the onset of vapor breakthrough for both the sorbent-packed column and the blank column prepared with uncoated glass beads. The blank column breakthrough time was subtracted from that for the sorbent to obtain the corrected breakthrough time of the sorbent. In each case, the breakthrough time was normalized to the mass of the sorbent for comparison to other samples.

## 3. Results

**Figure 2:**
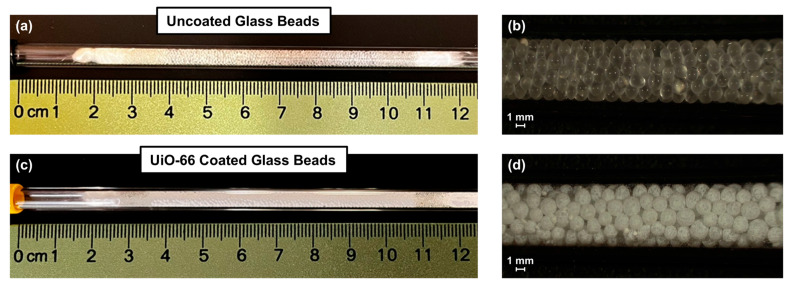
Photographs of a column prepared with uncoated glass beads (**a**) and a close-up image of the packed beads (**b**), as well as a column prepared with UiO-66 coated on glass beads without any binder (**c**) and a close-up image of the coated beads packed in the column (**d**).

The feasibility of coating MOF powders directly onto glass beads using adhesive forces was initially evaluated using UiO-66. Figure 2 shows photographs of the column of coated beads (c/d) compared to a reference column of uncoated beads (a/b). In the close-up images (b/d), a uniform coating of the MOF on the surface of the glass beads was visible, demonstrating successful coverage in the absence of binder. A few larger agglomerates of the MOF were also visible, which may have been too large to attach to the glass beads through adhesive forces alone.

**Figure 3:**
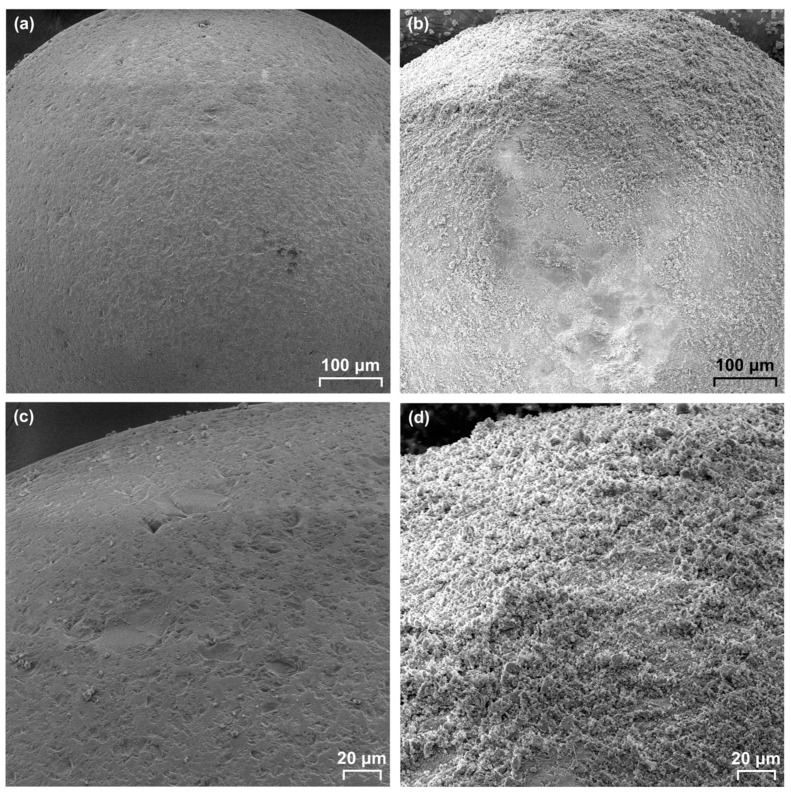
Scanning electron micrographs of (**a**) an uncoated glass bead at 1k magnification, (**b**) a glass bead coated with UiO-66 by adhesive forces at 1k magnification, (**c**) the uncoated bead at 3k magnification, and (**d**) the coated bead at 3k magnification.

Scanning electron micrographs of the coated and uncoated beads were collected to further investigate the UiO-66 coating. Figure 3a,c shows an uncoated glass bead at two different magnifications, and Figure 3b,d shows a glass bead coated with UiO-66. In micrographs of the uncoated bead, the relatively smooth surface of the glass was visible. After coating with UiO-66, the surface of the bead exhibited a rough and slightly uneven texture, consistent with a powder coating. Figure 3d also showed no particles larger than 10–20 μm, suggesting that the MOF particles are of a suitable size range for adhesion to the glass surface [30]. Together with the photographs in Figure 2, these images support successful coating of the beads.

To evaluate whether this sample preparation approach offers any experimental benefits, the breakthrough performance of this column was measured and compared to a similar column prepared using the binder trimethylolpropane triglycidyl ether as reported by Alhashem et al. [18]. All other aspects of the column were kept constant, including the size of the glass beads, the quantity of glass beads, the quantity of MOF sorbent, and the dimensions of the column. In both cases, the columns were challenged with 20% water vapor in nitrogen at a constant flow rate of 5 sccm (standard cubic centimeters), and the resulting data were evaluated to determine the uptake capacity and breakthrough time of each sample.

**Figure 4:**
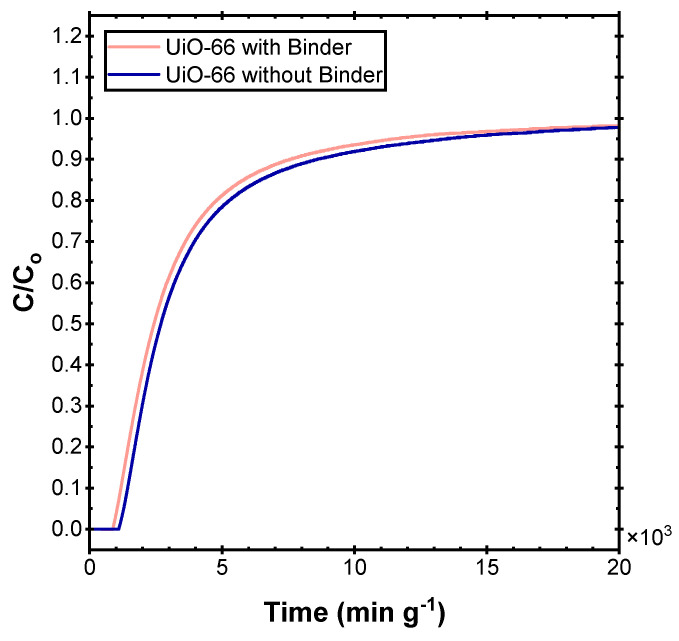
Breakthrough analysis of UiO-66 powder coated on glass beads with (pink) and without (dark blue) binder. The challenge gas was water vapor (20%) in nitrogen at 5 sccm.

As one might expect, the sample prepared using only adhesive forces (i.e., without binder) to coat the glass beads showed increased breakthrough performance relative to the sample prepared with binder (Figure 4). The breakthrough time increased to 1160 min g^−1^ versus 901 min g^−1^ for the sample prepared with binder. In addition, quantification of the total water adsorbed by each sample revealed that the column prepared using adhesive forces had an uptake capacity of 2.78 mmol g^−1^ compared to 2.35 mmol g^−1^ when the binder was used. In other words, the elimination of the binder yielded a 29% increase in breakthrough time and an 18% increase in uptake capacity. Of course, this is just one of many possible binders, and selection of a different binder such as polyvinyl alcohol/polyvinyl butyral [33] might also improve the sorbent performance. However, that approach would require optimization on a sample-by-sample basis to obtain the best performance from each one, since the same binder can have varying effects on different MOFs even within the same family (ex. UiO-66 and UiO-66-NH_2_) [19,33].

Additional experiments were performed to ensure that sample preparation could be carried out reproducibly (Table 1). First, a series of four columns (entries 1–4) was prepared under identical conditions to those described above for the initial experiments with UiO-66. From these columns, an average uptake capacity of 2.98 mmol g^−1^ was calculated with a standard deviation of 0.23 mmol g^−1^. This result agrees with the original uptake capacity of 2.78 mmol g^−1^. Moreover, these columns showed an average breakthrough time of 1160 ± 90 min g^−1^, which is consistent with the original column of UiO-66 prepared using adhesive forces.

Next, the quantity of UiO-66 in the column was varied (Table 1, entries 5–9) while keeping the quantity of glass beads constant. While both the uptake capacity and breakthrough time were normalized to the mass of the sorbet, it is possible that variations in packing of the powder in the column could still influence these values. This possibility is especially relevant at higher loadings, where the glass beads may become saturated and unbound powder may be introduced into the column. However, all of the samples tested—ranging from 9.84 to 48.3 mg of UiO-66—gave similar uptake capacities that were consistent with those found in entries 1–4 (2.98 ± 0.23 mmol g^−1^). In addition, nearly all of the samples gave breakthrough times that were consistent with those previously observed (1160 ± 90 min g^−1^). The only exception was the sample prepared with 9.8 mg UiO-66 (entry 9), which gave a breakthrough time of 1400 min g^−1^. It should be noted, though, that the next sample with 11.3 mg UiO-66 (entry 8) produced a lower breakthrough time (1160 min g^−1^) that is consistent with previous results. It is therefore believed that the breakthrough time in entry 9 is simply an outlier and is not representative of a meaningful deviation in measured properties, especially since its uptake capacity agrees with other experiments.

Instead, when the quantity of UiO-66 was kept constant and the quantity of glass beads was varied (Table 1, entries 10–12), more significant effects were observed. Increasing the quantity of beads from 1.0 g (entry 11) to 1.5 g (entry 10) resulted in an increase in uptake capacity from 3.08 mmol g^−1^ to 3.39 mmol g^−1^ and an increase in breakthrough time from 1170 min g^−1^ to 1510 min g^−1^. Both observations are consistent with the effects one might expect from making this change. Increasing the quantity of glass beads increases the bed depth, which in turn increases the contact time between the challenge gas and the MOF sorbent [14]. As such, it is not surprising that a column prepared with more glass beads would exhibit a higher capacity and longer breakthrough time.

When the quantity of glass beads was decreased from 1.0 g (entry 11) to 0.5 g (entry 12), a similar increase in uptake capacity (3.08 mmol g^−1^ to 3.51 mmol g^−1^, respectively) and breakthrough time (1170 min g^−1^ to 1350 min g^−1^, respectively) was observed. This result might seem surprising, since one might expect a column with fewer glass beads to exhibit a lower uptake capacity and breakthrough time. However, decreasing the quantity of glass beads to this degree also allowed a significant portion of uncoated MOF powder in the column due to oversaturation of the glass beads (Figure S13). As a result, the powder is able to compact within the interstitial space between the glass beads, likely leading to some back pressure and an artificial delay in the breakthrough data. Therefore, this result highlights that a sufficient quantity of glass beads must be used to completely immobilize the sorbent powder.

To test the stability of the sorbent coatings, a column was prepared under standard conditions (i.e., those used for entries 1–4) and subjected to 500 sccm of dry air for 96 h prior to measurement. This level of airflow was selected to represent an extreme case, as it was much higher than the airflow used in any of our measurements (5 sccm). Nonetheless, when the sample was measured by breakthrough analysis, its uptake capacity was in line with previous measurements (3.26 mmol g^−1^ vs. 2.98 ± 0.23 mmol g^−1^ for entries 1–4 in Table 2). Instead, its breakthrough time was slightly elevated (1330 min g^−1^ vs. 1160 ± 90 min g^−1^), possibly indicating movement of the sorbent particles under these conditions, though the effect was small (14% increase in breakthrough time) even at this high level of air flow.

**Figure 5:**
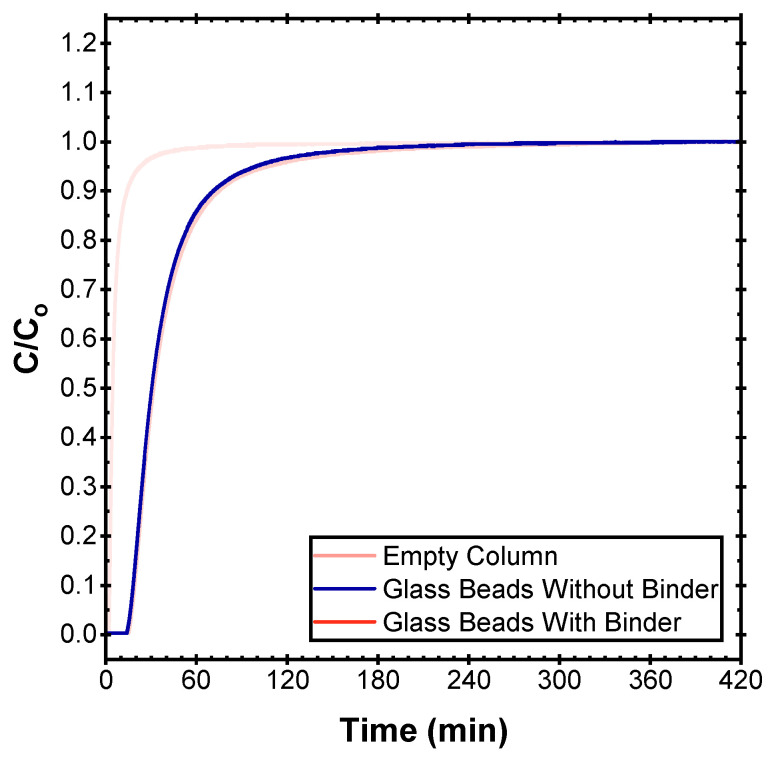
Control measurements on blank columns loaded with uncoated glass beads both with (red) and without (dark blue) binder, and comparison to an empty column (pink). The challenge gas was water vapor (20%) in nitrogen at 5 sccm.

Finally, a series of measurements was made on blank columns with and without glass beads to evaluate possible contributions of the beads to the measured data (Figure 5). While no effect was observed from the presence of binder, both columns containing glass beads (with and without binder) showed delayed breakthrough of water relative to an empty column. This observation was attributed to the fact that all measurements were preceded by a drying stage, in which the column was heated to 150 °C under dry nitrogen flow for 2 h. As a result, there was likely some degree of adsorption on the surface of the glass at the beginning of the experiment. To control for this effect, all data analysis reported herein was completed using a column of uncoated glass beads with a similar mass as a reference system.

**Figure 6:**
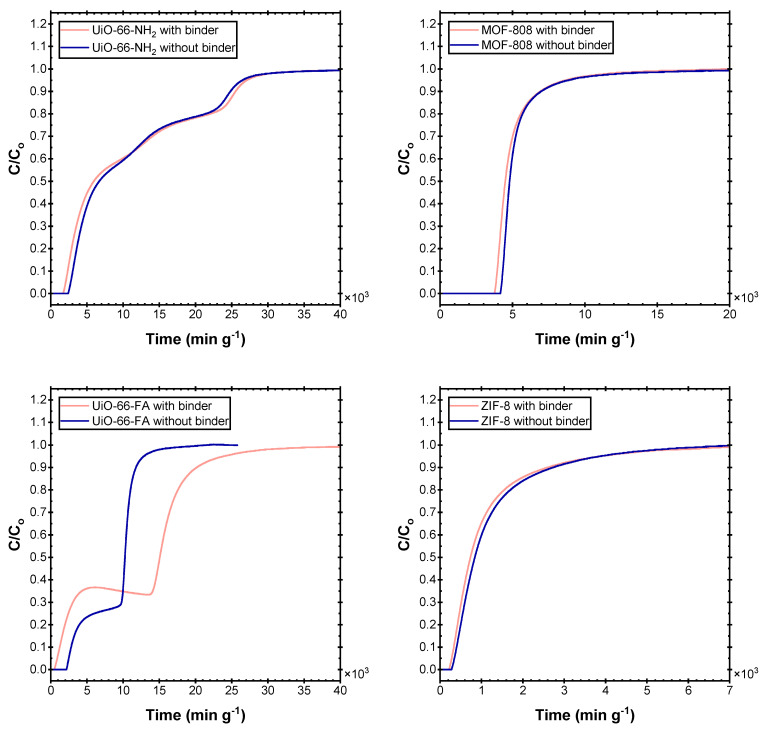
Breakthrough analysis of UiO-66-NH_2_ (**top left**), MOF-808-Ac (**top right**), UiO-66-FA (**bottom left**), and ZIF-8 (**bottom right**) coated on glass beads with binder (pink) and without binder (dark blue). The challenge gas was water vapor (20%) in nitrogen at 5 sccm.

To evaluate the applicability of this approach to other materials, sample columns were prepared using UiO-66-NH_2_, an amine-functionalized variant of UiO-66; UiO-66-FA, a derivative of UiO-66 with fumarate linkers instead of phthalate linkers; MOF-808; and ZIF-8 (Figure 6). Columns were once again prepared with and without binder to compare the effects from the binder versus the coating only using adhesive forces.

Similar to UiO-66, column breakthrough occurred 37% later for UiO-66-NH_2_ when it was coated onto glass beads using adhesive forces (2460 min g^−1^) rather than a binder (1800 min g^−1^), representing a significant increase in breakthrough performance. However, effectively no impact was observed on uptake capacity (Table 2, entries 16 and 17), which was 16.4 mmol g^−1^ for the sample without binder compared to 16.0 mmol g^−1^ when the binder was used. Analogously, only a small effect was observed for MOF-808 (Table 2, entries 18 and 19). The uptake capacity and breakthrough time of the sample prepared using adhesive forces were 8.03 mmol g^−1^ and 4230 min g^−1^, respectively. While these values are higher than for the sample prepared using binder (7.43 mmol g^−1^ and 3820 mmol g^−1^), they only represent a ~10% increase in breakthrough performance—significantly less than the 20–30% increase observed for UiO-66. In the case of MOF-808, the smaller effect may be due to differences in pore size, since the larger pores of MOF-808 (18 Å) may be less prone to clogging and occlusion than the smaller pores of UiO-66 (8 Å) [34]. This variation highlights an important consideration that additives such as binders may not have an equal impact on every material. In contrast, the use of adhesive forces allows for the analysis of pure materials, enabling measurement of their fundamental properties and direct comparisons of different sorbents without convoluting effects from other chemicals.

Interestingly, UiO-66-FA exhibited a higher uptake capacity when a binder was used (19.3 mmol g^−1^) versus when the coating was achieved using adhesive forces alone (12.5 mmol g^−1^). However, its breakthrough time was significantly reduced from 2180 min g^−1^ to 536 min g^−1^ when the binder was used. It should also be noted that the breakthrough curve for this sample showed a stepwise adsorption with decreasing water concentration in the effluent following the initial breakthrough event. It is not clear why this sample exhibited this behavior, but one hypothesis is a reaction between the binder and the alkene in the fumarate linker, though there is no evidence to support that hypothesis currently.

**Figure 7:**
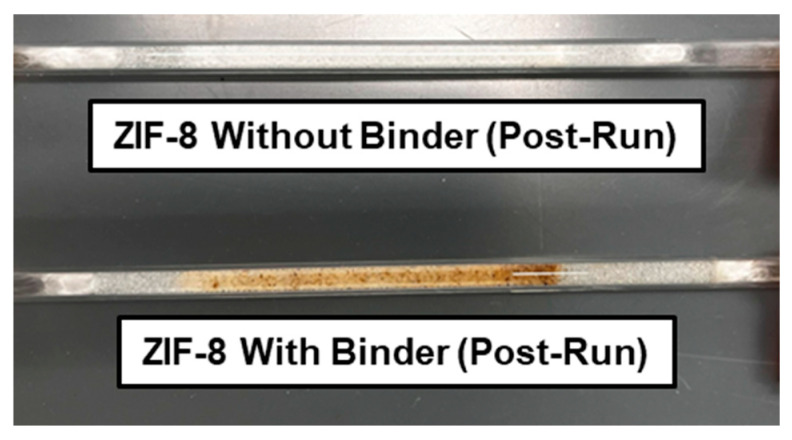
Photographs taken after breakthrough measurements of ZIF-8 sample columns prepared with (**bottom**) and without (**top**) binder.

**Figure 8:**
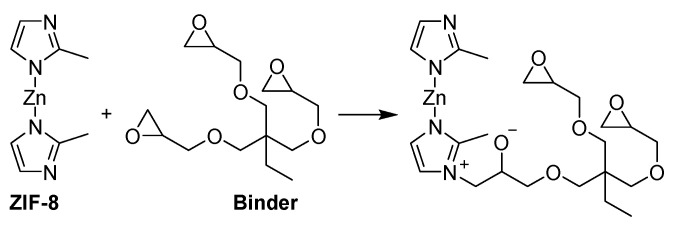
Proposed scheme for the reaction of ZIF-8 with the binder used in this work.

In contrast, when ZIF-8 was investigated, a color change was observed in the presence of the trimethylolpropane triglycidyl ether binder (Figure 7). While no change was initially observed during sample preparation, after the breakthrough measurement, the sample of ZIF-8 coated with binder changed from white to brown in color. This behavior was not observed for the sample coated using adhesive forces, and since both columns were otherwise subjected to identical conditions, this observation was attributed to the presence of the binder. A search of the literature revealed that a reaction between imidazoles and epoxides is possible [35,36,37,38,39], and that imidazoles have been used for curing epoxy resins for many years [37,38,39]. A scheme for this proposed reaction is shown in Figure 8, which involves an amine in the imidazole attacking the epoxide and causing its ring-opening. Regardless of the exact cause of this color change, the potential for this unintended reaction between the sorbent and the binder highlights another advantage of our simplified approach, since reducing the number of components reduces the potential for unintended chemical reactions.

## 4. Conclusions

This work has demonstrated a simple approach to preparing samples of sorbent such as MOFs for breakthrough analysis. Stable coatings of sorbents can be prepared on glass beads using the adhesive force between the glass and the sorbent powder, provided that the particle size of the sorbent is suitably small. For particles on the order of tens of microns or less, one can expect these adhesive forces to be larger than the gravitational forces on the particles, making stable coatings possible. Compared to similar samples prepared with a binder, samples prepared via this approach generally exhibit higher uptake capacities and longer breakthrough times that may be more representative of their actual properties. This approach also reduces the potential for sorbent degradation by eliminating the possibility for reactions with the binder, further improving the reliability of the measurement. While this study focused on water vapor as an analyte for simplicity, we see no reason that this approach cannot be extended to other gas and vapor-phase analytes, and ongoing work is exploring that possibility with success.

Importantly, it should be noted that this approach does have limitations that should be considered depending on the application. Notably, there is a limit to the quantity of sorbent powder that can be coated onto glass beads using adhesive forces alone. In this work, the limit we found was approximately 2–5 percent by weight. For applications where greater quantities of sorbent may be beneficial, the use of a binder may be necessary to increase sorbent loadings. Moreover, one can expect this approach to only be suitable for gas phase measurements where forces from the eluent on the sorbent particles are relatively small. Finally, this technique is intended to gain insight into sorbent performance under ideal, unimpeded conditions. However, real-world applications of sorbents often require shaping with pressure or a binder to form pellets and granules that can be incorporated into filters or other containers. For such applications, the approach reported here may not be optimal, since one might wish to understand the performance of the shaped sorbent. With all of that said, in a basic research setting where one desires to quickly evaluate the native, unaltered properties of a novel sorbent material, we believe that this procedure offers a solution to sample preparation that is simple, quick, and reproducible.

## Figures and Tables

**Table 1:** Control experiments testing column-to-column reproducibility, varying the quantity of sorbent, varying the quantity of glass beads, and investigating the impact of air flow on the stability of the sorbent coating in a packed column.

Entry	Variation	Uptake Capacity (mmol g^−1^) ^[a]^	Difference ^[b]^	Breakthrough Time (min g^−1^) ^[c]^	Difference ^[b]^
***Column-to-Column Reproducibility***					
1	Column 1 ^[d]^	3.08	+3%	1170	+1%
2	Column 2	2.69	−10%	1090	−6%
3	Column 3	3.23	+8%	1280	+10%
4	Column 4	2.91	−2%	1110	−5%
	*Average =*	*2.98 ± 0.23*	*Average =*	*1160 ± 90*	
***Quantity of Sorbent***					
5	48.3 mg UiO-66	2.68	−10%	1170	+1%
6	23.9 mg UiO-66 ^[d]^	3.08	+3%	1170	+1%
7	14.8 mg UiO-66	2.92	−2%	1160	0%
8	11.3 mg UiO-66	2.78	−7%	1160	0%
9	9.8 mg UiO-66	3.23	+8%	1400	+20%
***Quantity of Glass Beads***					
10	1.5 g GB	3.39	+14%	1510	+30%
11	1.0 g GB ^[d]^	3.08	+3%	1170	+1%
12	0.5 g GB	3.51	+18%	1350	+16%
***Coating Stability***					
13	500 sccm for 96 h ^[e]^	3.26	+9%	1330	+14%

^[a]^ Calculated as the area under the uptake curve relative to the area under the same curve for a blank column. ^[b]^ Difference relative to the average of the 4 identical columns at the top of this table. ^[c]^ Breakthrough time corrected for the instrument response, which was measured using a blank column and normalized to the mass of sorbent used in the measurement. ^[d]^ Indicates that the same sample was used in these entries. ^[e]^ Column was prepared under the same conditions as entries 1–4 and then subjected to 500 sccm of dry air 96 h prior to measurement.

**Table 2:** Measured uptake capacity and breakthrough time for each of the MOF sorbents tested with water vapor (20% in nitrogen at 5 sccm).

Entry	Material	Binder?	Uptake Capacity (mmol g^−1^) ^[a]^	Difference ^[b]^	Breakthrough Time (min g^−1^) ^[c]^	Difference ^[b]^
14	UiO-66	No	2.78	+18%	1160	+29%
15		Yes	2.35		901	
16	UiO-66-NH_2_	No	16.4	+3%	2460	+37%
17		Yes	16.0		1800	
18	MOF-808	No	8.03	+8%	4230	+11%
19		Yes	7.43		3820	
20	UiO-66-FA	No	12.5	−33%	2180	+307%
21		Yes	19.3		536	
22	ZIF-8 ^[d]^	No	1.05	+29%	297	+28%
23		Yes	0.812		233	

^[a]^ Calculated as the area under the uptake curve relative to the area under the same curve for a blank column. ^[b]^ Difference resulting from elimination of the binder. ^[c]^ Breakthrough time corrected for the instrument response, which was measured using a blank column, and normalized to the mass of sorbent used in the measurement. ^[d]^ Note that a reaction was observed for ZIF-8 when prepared using a binder.

## Data Availability

The original contributions presented in this study are included in the article/Supplementary Materials. Further inquiries can be directed to the corresponding author.

## Supplementary Materials

The following supporting information can be downloaded at: https://www.mdpi.com/article/10.3390/mps9030080/s1, Figures S1–S28.

## Distribution Statement

Approved for public release; distribution is unlimited.
