# Supplementary material for: A Simple Approach to Sample Preparation for Accurate and Reproducible Gas Phase Breakthrough Analysis of Adsorbent Materials
Source: Methods Protoc. 2026 May 29;9(3):80. doi: 10.3390/mps9030080 (PMC13304718; doi:10.3390/mps9030080)
Supplement: Supplementary file 1 [file mps-09-00080-s001.zip › mps-4254977-supplementary.pdf]

Supplementary Materials

for

A Simple Approach to Sample Preparation for Accurate and Reproducible Gas Phase Breakthrough Analysis of Adsorbent Materials

Daniel A. Corbin*, Christopher J. Breshike, Michael R. Papantonakis, R. Andrew McGill

US Naval Research Laboratory, 4555 Overlook Ave SW, Washington, DC 20375
*Corresponding Author: daniel.a.corbin.civ@us.navy.mil

Table of Contents

Control: Column-to-Column Reproducibility 2
Control: Quantity of Sorbent 4
Control: Quantity of Glass Beads 7
UiO-66 With and Without Binder 9
UiO-66-NH_2 With and Without Binder 10
MOF-808 With and Without Binder 11
UiO-66-FA With and Without Binder 12
ZIF-8 With and Without Binder 13
Example Process for Data Analysis 14

DISTRIBUTION STATEMENT A: Approved for public release; distribution is unlimited.

Control: Column-to-Column Reproducibility

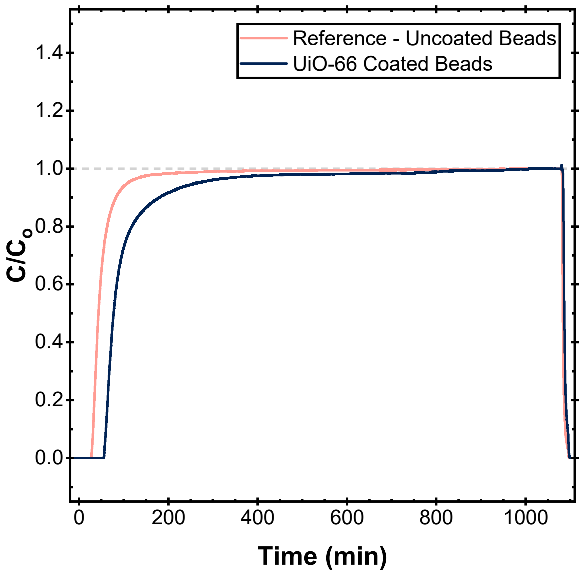

Figure S1. Breakthrough data (blue) testing column-to-column reproducibility. The reference curve collected using a column with uncoated glass beads is shown in pink. These data correspond to entry 1 in Table 1 of the main text and was prepared with 23.6 mg of UiO-66 and 1.0 g of glass beads.

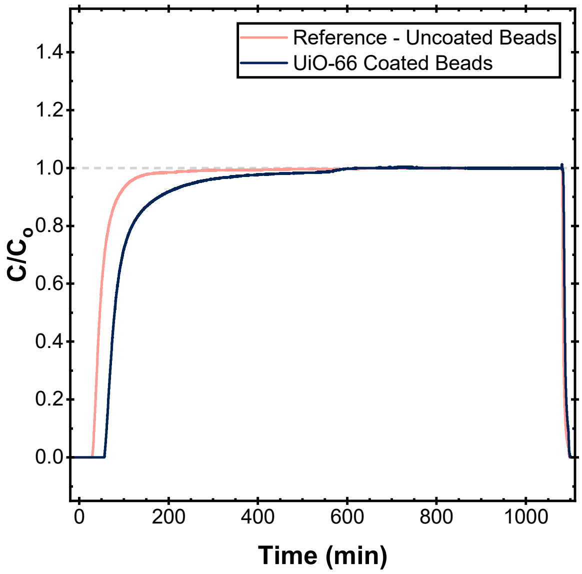

Figure S2. Breakthrough data (blue) testing column-to-column reproducibility. The reference curve collected using a column with uncoated glass beads is shown in pink. These data correspond to entry 2 in Table 1 of the main text and was prepared with 23.9 mg of UiO-66 and 1.0 g of glass beads.

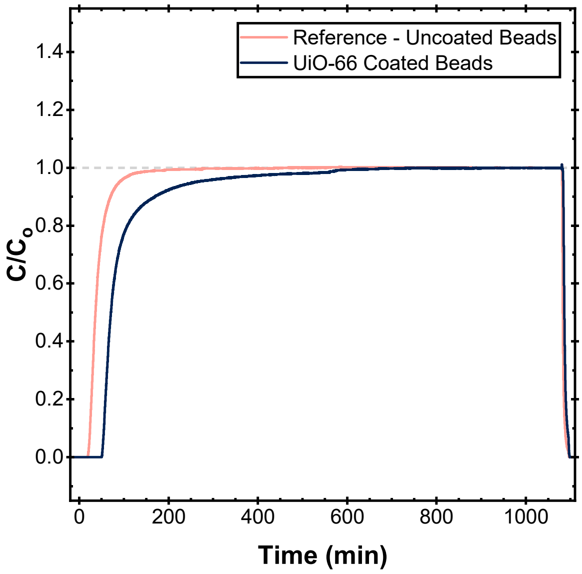

Figure S3. Breakthrough data (blue) testing column-to-column reproducibility. The reference curve collected using a column with uncoated glass beads is shown in pink. These data correspond to entry 3 in Table 1 of the main text and was prepared with 21.1 mg of UiO-66 and 1.0 g of glass beads.

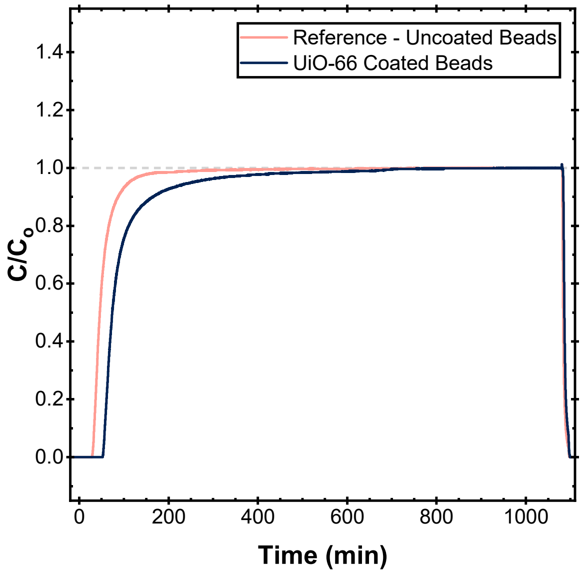

Figure S4. Breakthrough data (blue) testing column-to-column reproducibility. The reference curve collected using a column with uncoated glass beads is shown in pink. These data correspond to entry 4 in Table 1 of the main text and was prepared with 24.8 mg of UiO-66 and 1.0 g of glass beads.

Control: Quantity of Sorbent

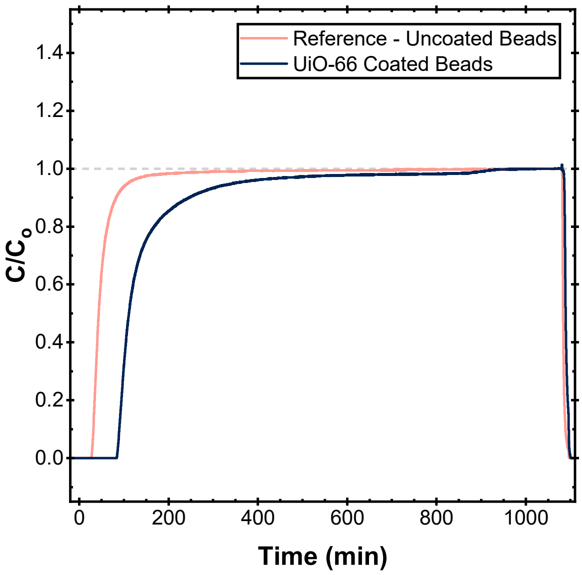

Figure S5. Breakthrough data (blue) testing the effect of the quantity of UiO-66 used in column preparation. The reference curve collected using a column with uncoated glass beads is shown in pink. These data correspond to entry 5 in Table 1 of the main text and was prepared with 48.3 mg of UiO-66 and 1.0 g of glass beads.

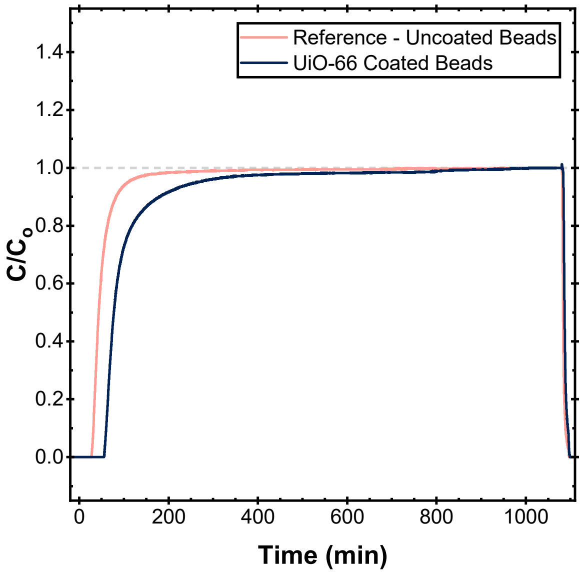

Figure S6. Breakthrough data (blue) testing the effect of the quantity of UiO-66 used in column preparation. The reference curve collected using a column with uncoated glass beads is shown in pink. These data correspond to entry 6 in Table 1 of the main text and was prepared with 23.9 mg of UiO-66 and 1.0 g of glass beads.

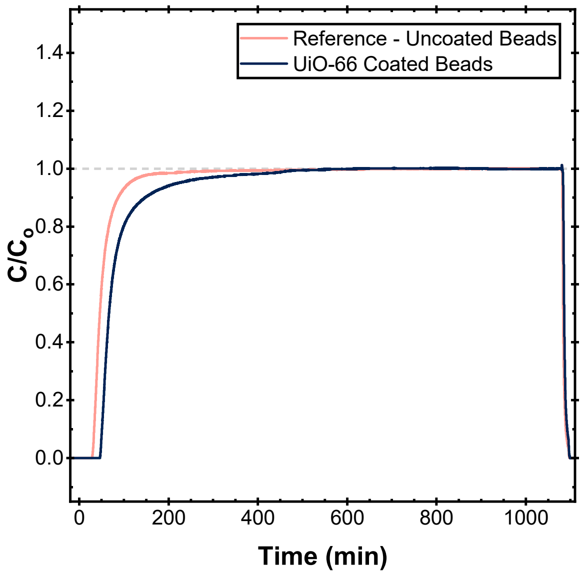

Figure S7. Breakthrough data (blue) testing the effect of the quantity of UiO-66 used in column preparation. The reference curve collected using a column with uncoated glass beads is shown in pink. These data correspond to entry 7 in Table 1 of the main text and was prepared with 14.8 mg of UiO-66 and 1.0 g of glass beads.

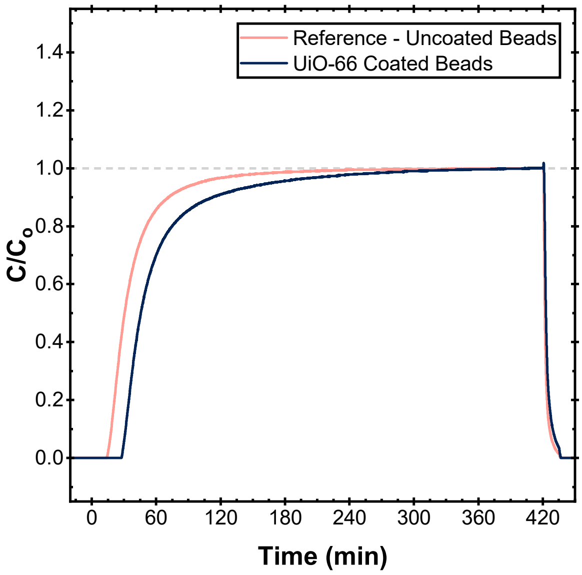

Figure S8. Breakthrough data (blue) testing the effect of the quantity of UiO-66 used in column preparation. The reference curve collected using a column with uncoated glass beads is shown in pink. These data correspond to entry 8 in Table 1 of the main text and was prepared with 11.3 mg of UiO-66 and 1.0 g of glass beads.

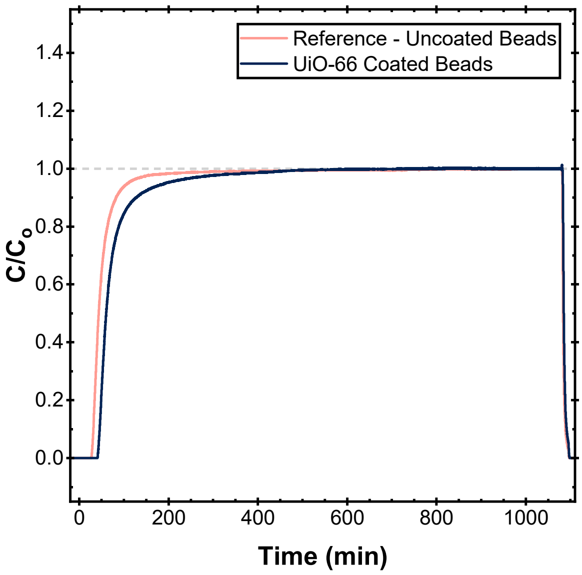

Figure S9. Breakthrough data (blue) testing the effect of the quantity of UiO-66 used in column preparation. The reference curve collected using a column with uncoated glass beads is shown in pink. These data correspond to entry 9 in Table 1 of the main text and was prepared with 9.8 mg of UiO-66 and 1.0 g of glass beads.

Control: Quantity of Glass Beads

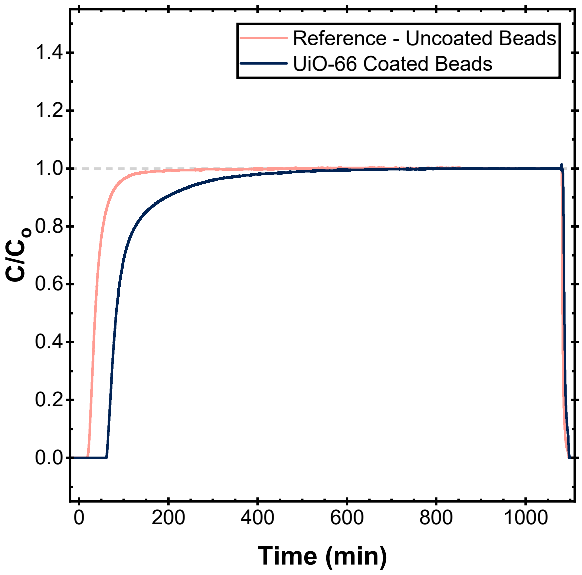

Figure S10. Breakthrough data (blue) testing the effect of the quantity of glass beads used in column preparation. The reference curve collected using a column with uncoated glass beads is shown in pink. These data correspond to entry 10 in Table 1 of the main text and was prepared with 27.1 mg of UiO-66 and 1.5 g of glass beads.

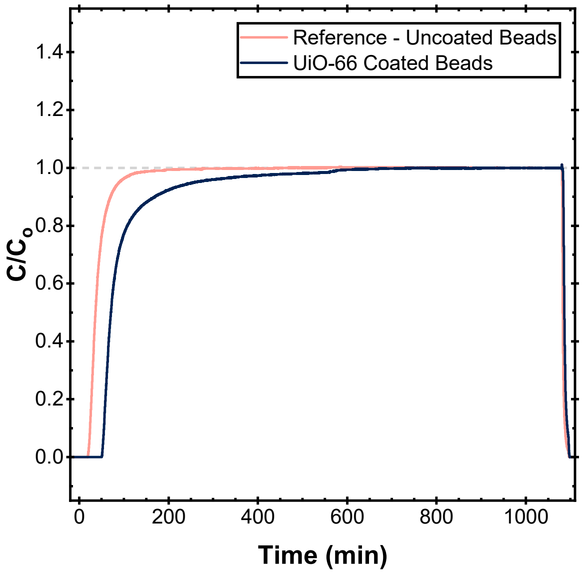

Figure S11. Breakthrough data (blue) testing the effect of the quantity of glass beads used in column preparation. The reference curve collected using a column with uncoated glass beads is shown in pink. These data correspond to entry 11 in Table 1 of the main text and was prepared with 23.6 mg of UiO-66 and 1.0 g of glass beads.

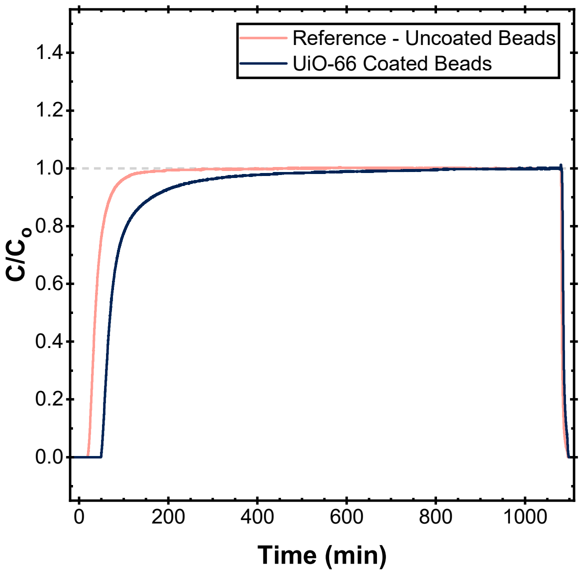

Figure S12. Breakthrough data (blue) testing the effect of the quantity of glass beads used in column preparation. The reference curve collected using a column with uncoated glass beads is shown in pink. These data correspond to entry 12 in Table 1 of the main text and was prepared with 21.3 mg of UiO-66 and 0.5 g of glass beads.

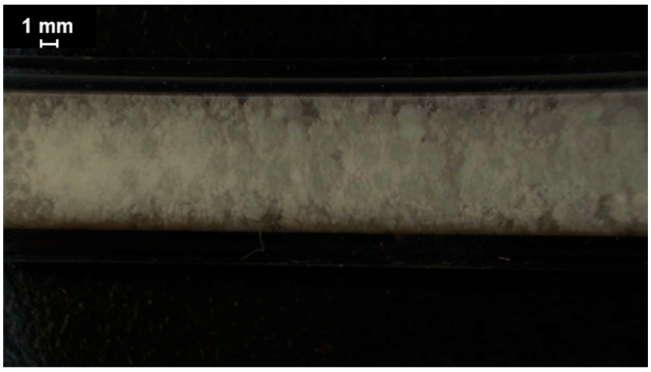

Figure S13. Photograph of the column measured to collect the data for entry 12 in Table 1 of the main text showing a significant portion of unbound MOF powder in the interstitial space between glass beads.

UiO-66 With and Without Binder

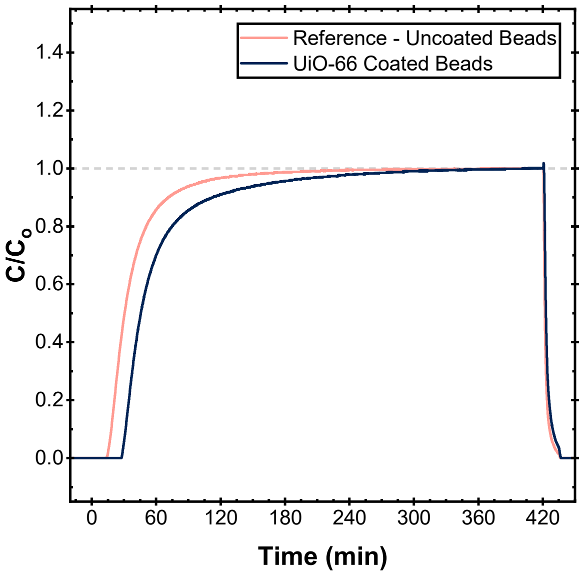

Figure S14. Breakthrough data (blue) for UiO-66 coated on glass beads using adhesive forces. The reference curve collected using a column with uncoated glass beads is shown in pink. These data correspond to entry 13 in Table 2 of the main text and was prepared with 11.3 mg of UiO-66 and 1.0 g of glass beads.

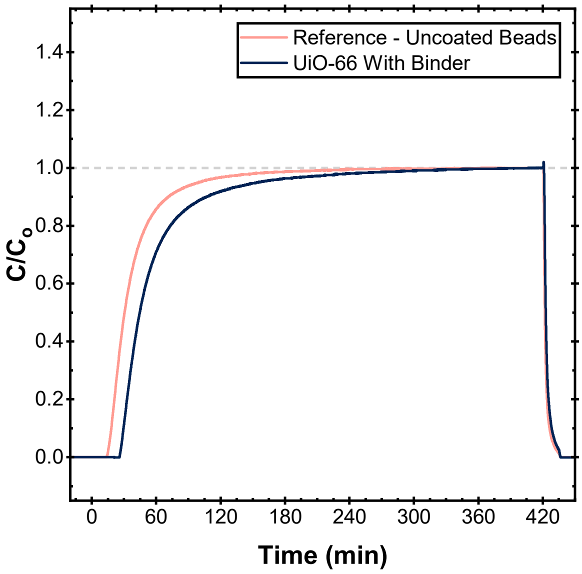

Figure S15. Breakthrough data (blue) for UiO-66 coated on glass beads using binder. The reference curve collected using a column with uncoated glass beads is shown in pink. These data correspond to entry 14 in Table 2 of the main text and was prepared with 12.1 mg of UiO-66 and 1.0 g of glass beads.

UiO-66-NH2 With and Without Binder

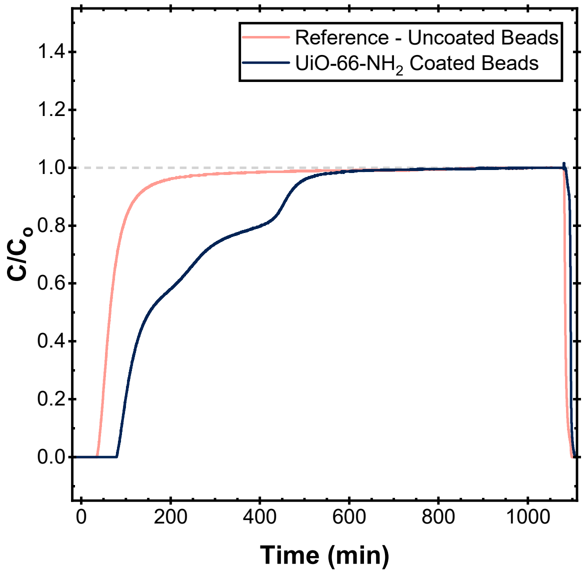

Figure S16. Breakthrough data (blue) for UiO-66-NH_2 coated on glass beads using adhesive forces. The reference curve collected using a column with uncoated glass beads is shown in pink. These data correspond to entry 15 in Table 2 of the main text and was prepared with 17.1 mg of UiO-66-NH_2 and 1.0 g of glass beads.

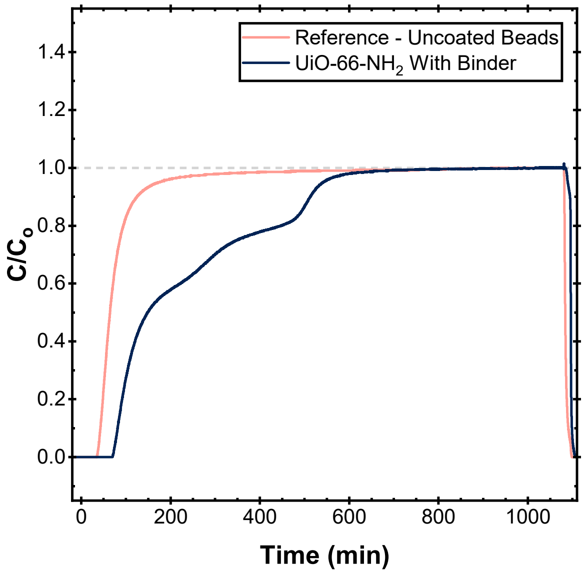

Figure S17. Breakthrough data (blue) for UiO-66-NH_2 coated on glass beads using binder. The reference curve collected using a column with uncoated glass beads is shown in pink. These data correspond to entry 16 in Table 2 of the main text and was prepared with 18.6 mg of UiO-66-NH_2 and 1.0 g of glass beads.

MOF-808 With and Without Binder

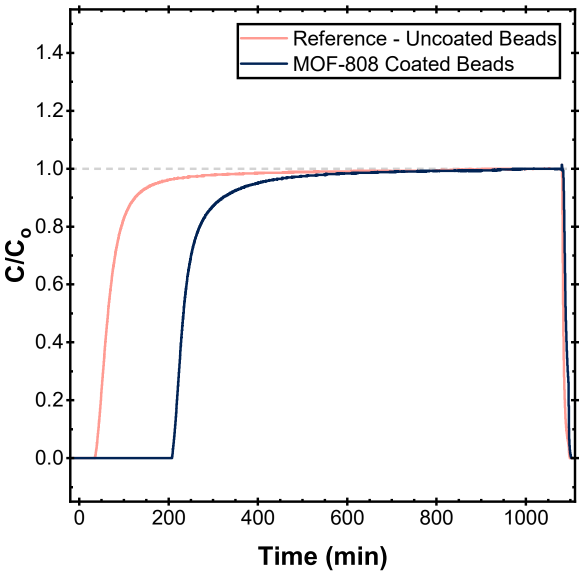

Figure S18. Breakthrough data (blue) for MOF-808 coated on glass beads using adhesive forces. The reference curve collected using a column with uncoated glass beads is shown in pink. These data correspond to entry 17 in Table 2 of the main text and was prepared with 40.7 mg of MOF-808 and 1.0 g of glass beads.

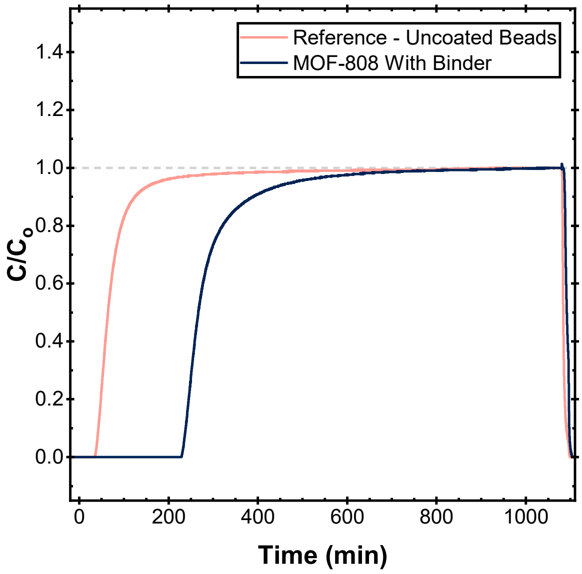

Figure S19. Breakthrough data (blue) for MOF-808 coated on glass beads using binder. The reference curve collected using a column with uncoated glass beads is shown in pink. These data correspond to entry 18 in Table 2 of the main text and was prepared with 50.8 mg of MOF-808 and 1.0 g of glass beads.

UiO-66-FA With and Without Binder

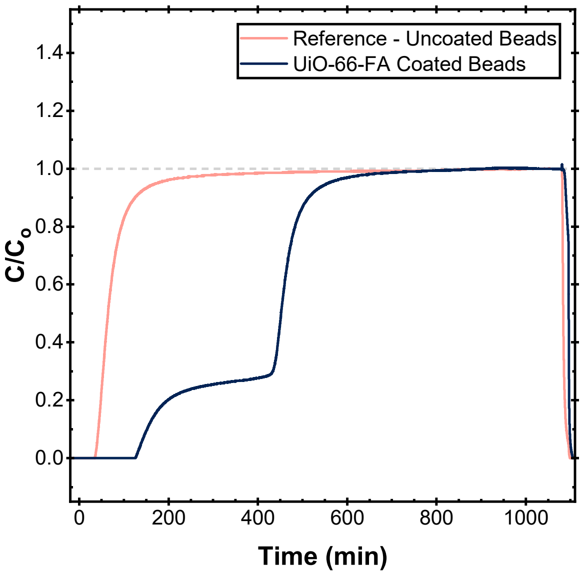

Figure S20. Breakthrough data (blue) for UiO-66-FA coated on glass beads using adhesive forces. The reference curve collected using a column with uncoated glass beads is shown in pink. These data correspond to entry 19 in Table 2 of the main text and was prepared with 40.4 mg of UiO-66-FA and 1.0 g of glass beads.

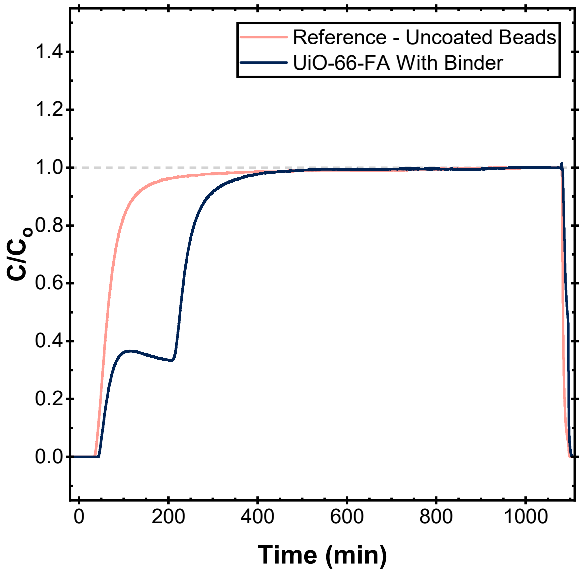

Figure S21. Breakthrough data (blue) for UiO-66-FA coated on glass beads using binder. The reference curve collected using a column with uncoated glass beads is shown in pink. These data correspond to entry 20 in Table 2 of the main text and was prepared with 12.5 mg of UiO-66-FA and 1.0 g of glass beads.

ZIF-8 With and Without Binder

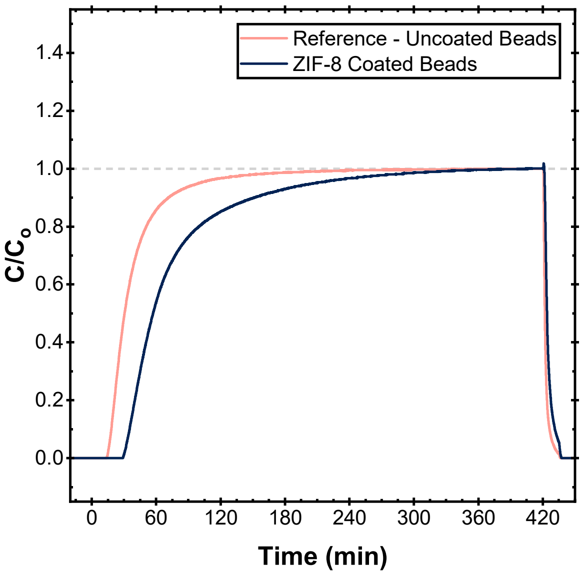

Figure S22. Breakthrough data (blue) for ZIF-8 coated on glass beads using adhesive forces. The reference curve collected using a column with uncoated glass beads is shown in pink. These data correspond to entry 21 in Table 2 of the main text and was prepared with 49.8 mg of ZIF-8 and 1.0 g of glass beads.

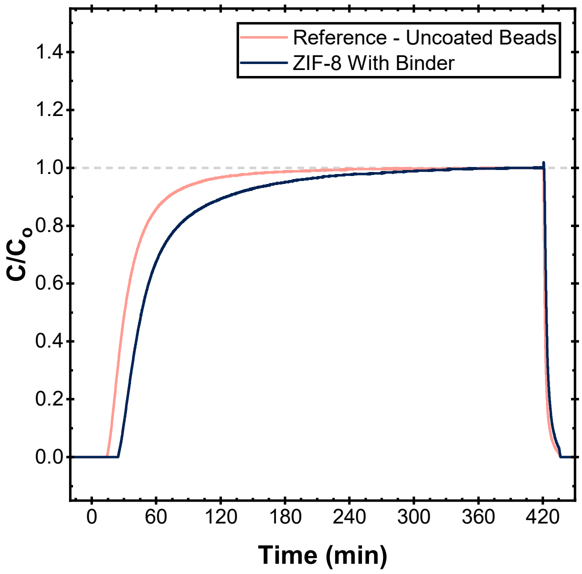

Figure S23. Breakthrough data (blue) for ZIF-8 coated on glass beads using binder. The reference curve collected using a column with uncoated glass beads is shown in pink. These data correspond to entry 22 in Table 2 of the main text and was prepared with 42.4 mg of ZIF-8 and 1.0 g of glass beads.

Example Workflow for Data Analysis

In a typical experiment, data from the breakthrough measurement are obtained as relative humidity (%) versus time. Figure S24 shows a plot of the data for an example experiment (entry 3 of Table 1).

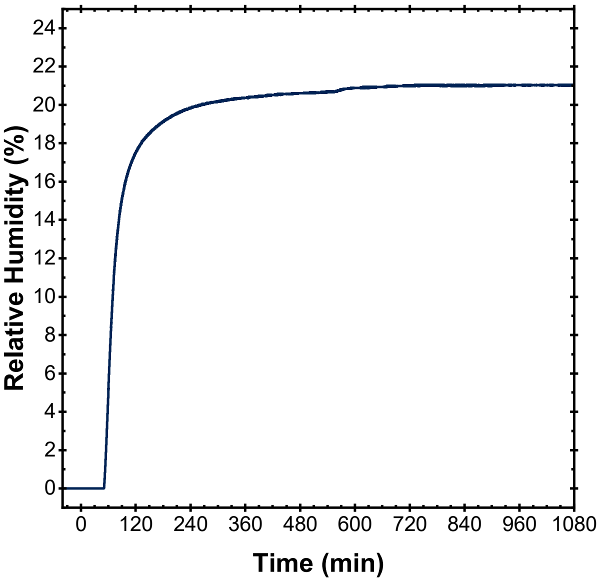

Figure S24. Plot of the raw data from a breakthrough measurement (20% water vapor in nitrogen at 5 sccm).

The data are first processed by applying a baseline correction and normalizing to the end of the curve, which typically corresponds to the maximum of the curve. To account for noise, the data for each one were averaged over a 20 min period (−20 min to 0 min for the baseline correction, and 1060 min to 1080 min for normalization). In other words, the baseline correction was applied by subtracting the average value of the baseline from the 20 minutes preceding the experiment, after which the data were normalized by dividing by the average signal from the last 20 minutes of the experiment. The result is shown in Figure S25.

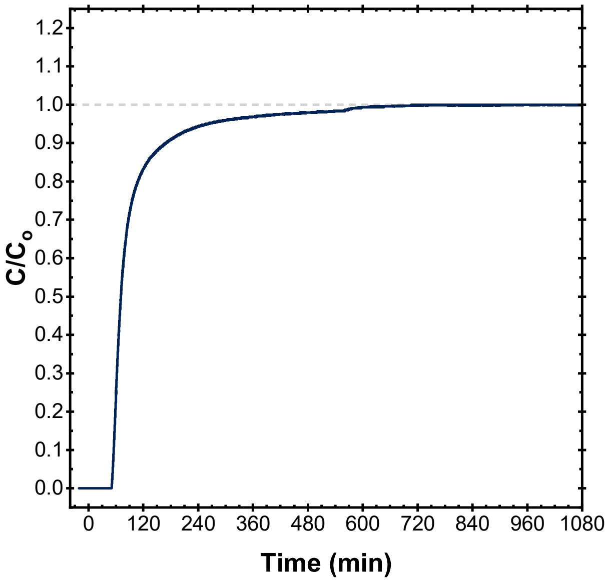

Figure S25. Plot of the data after baseline correction and normalization.

To determine the uptake capacity of the material, the data were integrated to obtain the area under the curve (Figure S26, dark blue). This area represents the vapor that was not adsorbed by the sorbent. The same was carried out for a reference curve of uncoated glass beads (Figure S26, pink + dark blue), representing the total quantity of vapor introduced in the experiment. The difference in area under the two curves (Figure S26, pink) corresponds to the quantity of vapor adsorbed by the sorbent being measured.

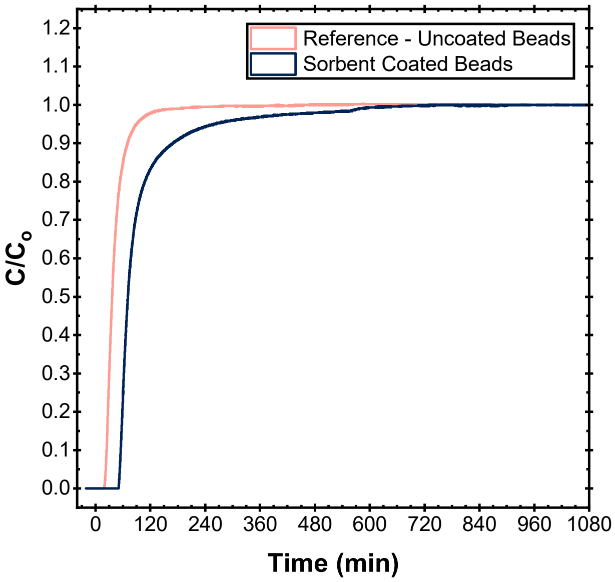

Figure S26. Plot demonstrating the different areas integrated for the calculation of uptake capacity.

In this example, the area of the reference curve is 1035.38315, while the area under the sorbent curve is 978.29096. The difference, corresponding to the area of the adsorbed vapor, is 57.09219 (5.51%). To convert this value into a useful quantity of the challenge vapor, we must first calculate how much of the vapor was introduced over the course of the experiment. In this case, the challenge vapor was water, which has a saturated vapor pressure of 23.8 mmHg at 25 °C (the temperature of the experiment). This corresponds to a saturated vapor concentration of 31316 ppm, or 23.0 g m^{-3}.

The actual challenge vapor stream had a relative humidity of 20%, which was achieved by mixing 1 sccm of the saturated water vapor with 4 sccm dry nitrogen. As such, the actual concentration of water in the vapor stream was 4.61 g m^{-3}, or 4.61 x 10^{-6} g cm^{-3}. Since the sorbent was exposed to the vapor stream for 1080 min at a constant flow rate of 5 sccm, the total volume of the challenge vapor was 5400 cm^3. Therefore, the total mass of water introduced in the experiment was 0.0249 g. Since we know that 5.51% of this water was adsorbed by the sorbent, this corresponded to 0.00137 g of the water being adsorbed (0.0761 mmol). In this case, 0.0236 g of sorbent was present in the column, giving an uptake capacity of 3.23 mmol g^{-1}.

To determine the breakthrough time of the material, the onset of the breakthrough curve was estimated as shown in Figure S27. The x-value at which the onset occurred was recorded, and the same process was repeated for the reference curve. For the example system described above, breakthrough for the sorbent occurred at 51.6 min, and breakthrough for the reference column occurred at 21.5 min. The reference breakthrough time was subtracted from the sorbent time to correct for the instrument response time, which includes factors such

as column dead volume, time to reach vapor saturation, and any adsorption on the inside of the column, the glass beads, or the instrument tubing. For this example, the corrected breakthrough time was 30.1 min, which was normalized to the mass of the sorbent (0.0236 g) for comparison to other samples. The final value was 1280 min g^{-1}.

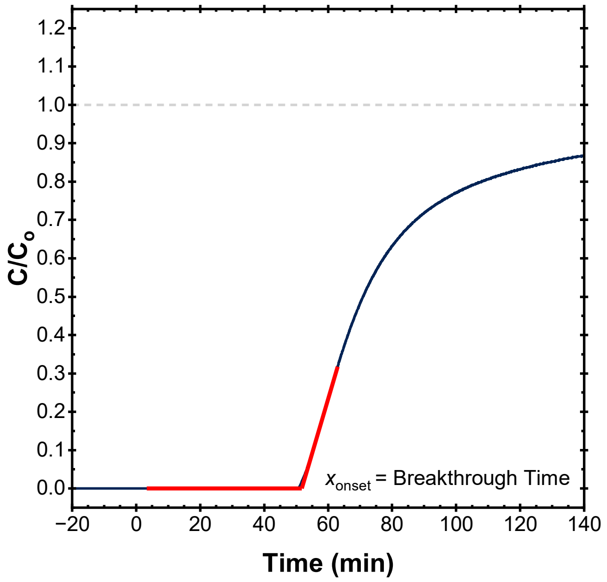

Figure S27. Estimation of the onset of breakthrough for the example sorbent.

For figures in the main text that compare two samples, these data also had to be normalized to the mass of sorbent in each experiment. In each case, the x-axis (time) was first corrected by subtracting the reference breakthrough time (21.5 min in the example above), followed by dividing by the mass of the sorbent (0.0236 g). The resulting data could be plotted with the x-axis (time) in units of min g^{-1}, allowing for an accurate comparison of samples measured with different quantities of sorbent. An example of the resulting data is shown in Figure S28.

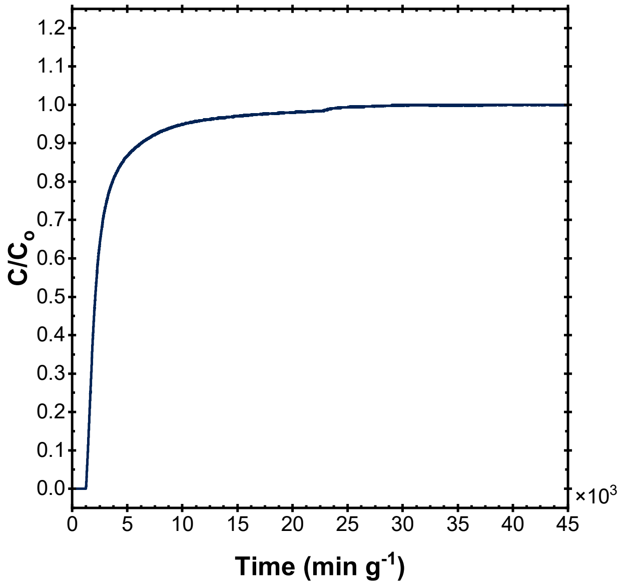

Figure S28. Example of data plotted after normalizing the x-axis to the mass of the sorbent.
